# Repairing Artifacts in Neural Activity Recordings Using Low-Rank Matrix Estimation

**DOI:** 10.3390/s23104847

**Published:** 2023-05-17

**Authors:** Shruti Naik, Ghislaine Dehaene-Lambertz, Demian Battaglia

**Affiliations:** 1 Cognitive Neuroimaging Unit, Centre National de la Recherche Scientifique (CNRS), Institut National de la Santé et de la Recherche Médicale (INSERM), CEA, Université Paris-Saclay, NeuroSpin Center, F-91190 Gif-sur-Yvette, France; 2 Institut de Neurosciences des Systèmes, U1106, Centre National de la Recherche Scientifique (CNRS) Aix-Marseille Université, F-13005 Marseille, France; 3 Institute for Advanced Studies, University of Strasbourg, (USIAS), F-67000 Strasbourg, France

**Keywords:** EEG, MEG, LFP, artifact correction, preprocessing, sensor noise, human infants

## Abstract

Electrophysiology recordings are frequently affected by artifacts (e.g., subject motion or eye movements), which reduces the number of available trials and affects the statistical power. When artifacts are unavoidable and data are scarce, signal reconstruction algorithms that allow for the retention of sufficient trials become crucial. Here, we present one such algorithm that makes use of large spatiotemporal correlations in neural signals and solves the low-rank matrix completion problem, to fix artifactual entries. The method uses a gradient descent algorithm in lower dimensions to learn the missing entries and provide faithful reconstruction of signals. We carried out numerical simulations to benchmark the method and estimate optimal hyperparameters for actual EEG data. The fidelity of reconstruction was assessed by detecting event-related potentials (ERP) from a highly artifacted EEG time series from human infants. The proposed method significantly improved the standardized error of the mean in ERP group analysis and a between-trial variability analysis compared to a state-of-the-art interpolation technique. This improvement increased the statistical power and revealed significant effects that would have been deemed insignificant without reconstruction. The method can be applied to any time-continuous neural signal where artifacts are sparse and spread out across epochs and channels, increasing data retention and statistical power.

## 1. Introduction

Electrophysiology recordings are valuable tools for studying the neural correlates of cognition in humans as well as animals. Despite the development of new imaging techniques such as fMRI and near-infrared spectroscopy, EEG and the related event related potentials (ERPs) remains among the most reliable and clinically feasible tools for studying cognition, especially in human populations, such as patients with pathology, elderly subjects, infants, and children [1,2].

However, one of the biggest challenges with EEG/MEG data is the very small signal-to-noise ratio, due to the wide range of sources of noise and artifacts. This problem is exacerbated in infants, where apart from line noise and faulty channels, unexpected movements and high amplitude voltage fluctuations are the norm rather than an exception. On the other hand, long and continuous data recording is difficult due to their low attention spans, fatigue, and frequent sleep episodes at this age. These problems result in a very high amount of trial rejections in infant studies. For example, it is common to have 100–300 trials per experimental condition in healthy adults, while in children and infants, usable trials can be as few as 30 or less. Any algorithm that can roughly reconstruct these lost signals to some extent becomes extremely valuable, in order to avoid rejecting too many trials, especially when they are so precious because they are so few in number, as in the case of infant experiments [3,4].

A common practice in current electrophysiology analyses is to detect possible noise sources either manually or using automated artifact detection algorithms, reject the noisy trials, and possibly repair (or interpolate) signals if the contamination of individual trials is minimal [5,6]. Since many of the current analysis paradigms (such as event related potentials (ERPs), event related fields (ERFs), event-related desynchronizations (ERD), and synchronization (ERS) of oscillatory activity, etc. [1,7]) rely on the averaging of signals across trials and across subjects, they require a large number of trials to yield robust and significant findings. One may hence want to increase the amount of trials by relaxing the criteria for artifacted trial exclusion; however, this would further increase the noisiness of the data. As a result, it is necessary to find a balance between the amount of noise included in evoked activation analysis and the number of retained trials. There would be a huge number of rejected trials if the artifact rejection procedure used with typical healthy adult populations was applied to atypical populations. Therefore, relaxed rejection criteria are used, resulting in the inclusion of time series with many more sporadic artifact-related “gaps” than in standard studies. This situation reveals an even more pressing need for an artifact rejection and repair algorithm.

Independent component analysis (ICA) and signal space projections (SSP) are common candidates for artifact detection, rejection, and/or repair in healthy adults [8,9,10,11]. These techniques remove noise by separating the common known sources of contamination, such as heartbeats, ocular movements due to eye blinks, and muscle movements. Since these artifacts are periodic, have a single noise source, and often include high amplitude fluctuations, they are easy to detect using ICA or SSP. However, large voltage fluctuations of neural origin may be more difficult to separate from fluctuations due to other environmental or biological noise sources. Such high-amplitude fluctuations are more common in the recordings of atypical populations than of healthy populations [12] and in infants relative to adults, due to poor skull ossification and brain immaturity. Reference [5] showed, for example, that ICA does not improve ERP robustness in young infants. Moreover, these algorithms cannot correct for the motion artifacts that are very frequent and randomly distributed throughout an experiment. A common practice in adults is to simply omit the epochs containing motion artifacts based on manual inspection before and after ICA or SSP has been applied. The resulting gaps still have to be fit by a suitable artifact repair algorithm.

Here, we propose a novel artifact repair method that is agnostic to the sources of artifacts. We consider the task of artifact repair as a *matrix completion* problem, which is a well-studied machine-learning task of filling in the missing entries from a partially observed matrix (here the channel × time matrix of neural activity observations) [13]. The algorithm we propose here is called OPTSPACE, which was first proposed for use in recommender systems, such as to reveal the user preferences [14]. Such systems try to predict the items (e.g., a Netflix show) that a user might like on the basis of the user’s ratings of similar items, as well as from the reactions of similar users [15]. Theoretical proofs and numerical solutions have been provided for this algorithm, showing that the missing entries of such a matrix (in our case, due to artifact corruption) can be recovered with very high accuracy, given specific bounds on the revealed matrix entries, and with the assumption that the original matrix has low dimensionality, yielding successful applications in the fields of collaborative filtering, compressed sensing, and image processing [14,16,17].

Here, we tested whether a similar algorithm can be applied to neural data and, specifically, to repair EEG artifacts from recordings of human infants. The precise problem is as follows: Given multivariate time-series data (from 128 channels), if there exists moments where some of the channels are corrupted, can we recover these entries making use of the spatiotemporal correlations? Hence, after all the typical preprocessing steps were performed for artifact detection, we applied the proposed learning algorithm to filling-in the remaining artifact-related gaps. For the use of this algorithm with neural data, we first needed to ensure that the neural data met the requirements, in terms of the assumptions of the original algorithm. One of the first assumptions is that the matrix to be filled has low-dimensional representation or a low rank. This can happen if the time-series data have a large degree of local linear interdependency (over time), or when neural state trajectories unfold over manifolds of a lower dimension than the number of available channels. Precisely this hypothesis has recently been advocated by an impressive number of independent theoretical and experimental system neuroscience studies at different spatiotemporal scales [18,19,20,21,22,23,24,25,26,27]. The low-dimensionality of neural recordings has been explicitly quantified for several neural datasets [28,29]. Especially in the case of high-density EEG data, the underlying brain sources generating scalp topography are much fewer than the number of sensors [30,31]. Therefore, we considered it legitimate to base our artifact repair procedure on a powerful low-rank matrix completion algorithm, which treats corrupted signal observations as "missing values" to be predicted based on their interdependence with other observed entries.

As neural data have a very different structure from the algorithm’s original use in collaborative filtering, we decided to benchmark the algorithm, to ensure that the application of this algorithm to neural data was robust. Specifically, we studied the algorithm performance in two ways. First, we benchmarked the current approach by testing its performance on data with artificially created “gaps” that preserved the exact artifact patterns of a typical EEG dataset. Since the ground truth data hidden by the artifacts were known for these realistic surrogate data, we could quantify how accurately the algorithm could recover these signals, as a function of the severity of the applied artifacts. Second, to test the usefulness of such an approach for the analyses in real cognitive neuroscience experiments, we demonstrated that this gap-filling algorithm could significantly improve the statistical power in the detection of ERP components, as compared to spherical spline interpolation [32], a state-of-the-art competitor algorithm. Moreover, we also showed that our method allowed for a better comparison of single-subject metrics, such as between-trial variability. In particular, thanks to an improved number of trials after the signal repair, we were able to prove the existence of significant differences in between-trial variability across young and older infants, which had previously been studied [33] but deemed not significant, because of the large number of artifacted and non-repaired trials.

## 2. Materials and Methods

### 2.1. Proposed Algorithm and Assumptions

Let **D** be a C×T neural data matrix, where *C* represents the total number of channels and *T* the length of the signal time series. We assume that this large matrix **D** has already been examined for artifact detection and contains missing entries for which data has been marked as corrupted and not recoverable using more conventional techniques. We present an algorithm for filling these entries that is based on the matrix completion algorithm known as OptSpace introduced by [14]. For full details of the matrix completion algorithm itself and proofs of its convergence, we refer to the original literature. However, in this section we will explain in a simplified manner the essence of its operation and how we use it to specifically perform multi-variate time-series reparation.

The first step of our approach is *data epoching*, i.e., the usually large data matrix **D** is first split into a finite number of short nonoverlapping epochs $M_{k}$ of size $C\times T_{k}$ ($T_{k}<<T$). The reason for data epoching is twofold. First, applying a matrix completion algorithm to the entire data matrix at once is computationally expensive and sometimes impossible, due to memory constraints, whereas dividing it into shorter epochs can achieve a faster convergence. Second, epoching enables temporally local prediction of the missing entries, by employing different optimized projections for different epochs, as we will see later. It should be noted, however, that this data epoching procedure differs from the epoching process of the event-related paradigm. Unlike ERP epochs, in this step, we are agnostic to the external events, i.e., stimulus onsets. We simply divide the full-length continuous time-series matrix into nonoverlapping smaller blocks.

The next step consists of applying a *matrix completion* procedure epoch by epoch. Specifically, for each such small matrix M, let Ω(M) be the set of all known (noncorrupted) entries of a given data epoch and ℧(M) its complement, i.e., the set of all corrupted entries. To fill the gaps in M we need to infer values for the entries ℧(M) solely based on the entries in Ω(M) and from the assumption that the original matrix M can be approximated using a reasonably low rank matrix with rank *r*, i.e., $M_{\mathrm{ij}}={\hat{M}}_{\mathrm{ij}}+Z_{\mathrm{ij}}$, where $\hat{M}={\mathrm{XSY}}^{T}$ and Z contains a deviation from such a low rank matrix $\hat{M}$, due to noise. Here, we use the ansatz, reflecting our low-rank hypothesis, that the matrix component M can be decomposed in terms of the three matrices X, S, and Y, where S is a diagonal matrix of singular values and $X\in R^{C\times r}$ and $Y\in R^{T_{k}\times r}$ provide approximate projections of the original data epochs onto a hyperplane spanned by *r* orthogonal directions.

**Figure 1:**
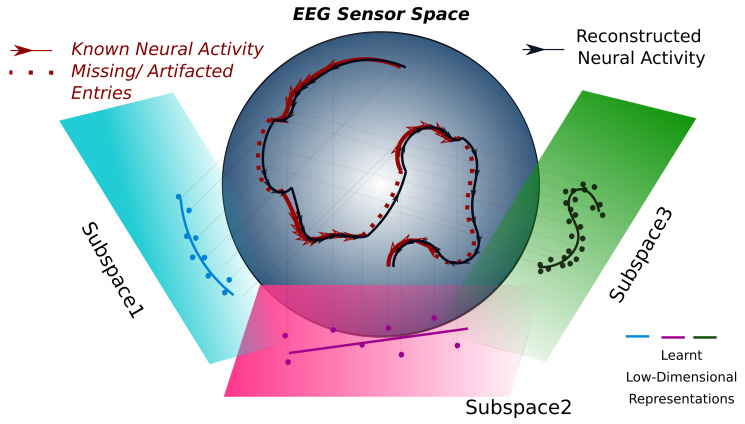
Schematic explanation of the proposed method. Multi-sensor neural data can be considered a trajectory in a high-dimensional space. However, the knowledge of this trajectory is incomplete, as some observations are missing, generally because of artifacts corrupting them (as indicated by the dashed red line sections in the figure here). We can, however, make the hypothesis that the trajectory is continuous and unfolds over a low-dimensional manifold in data space (here represented as a sphere). Under these assumptions, we can model short segments of the trajectory via their projections on locally optimized hyperplanes, with the same dimensionality of the actual unknown manifold. Through the optimization procedure, an inference is performed for the whole trajectory segment projection, i.e., for the observed but also the unobserved data points. In this way, reasonable guesses for discarded data segments can be obtained, in a way respectful of the local data geometry. This figure also serves as a graphical abstract.

This step is reminiscent of other dimensionality reduction approaches, in which the signal is decomposed in terms of the time-courses of a discrete number of factors (e.g., in ICA, the time-courses of a few spatial components of interest or common sources of a signal). However, here, the factors are optimized epoch by epoch, unlike in conventional dimensionality reduction approaches, which are usually performed over the whole data matrix D or only on the epochs of relevant trials. Mathematically, this means that we model the original neural trajectories unfolding on a generally nonlinear *r*-dimensional manifold in $R^{C}$ as a trajectory on the linear Grassmanian manifold $\mathrm{Gr}\left(r,R^{C}\right)$ of the full-dimensional data space $R^{C}$. This somewhat esoteric jargon hides a construction that is relatively simple to visualize (cf. Figure 1, which also serves as a graphical abstract). Intuitively, the procedure tries to approximate the original trajectory by sampling from the linear projections on a series of flat hyperplanes, whose orientation is locally optimized epoch by epoch.

Going back to the matrix completion algorithm, for each of the blocks M, we could determine its missing entries by choosing an optimal projection space, such that the following cost function is minimized:

$$F\left(X,Y\right)=\min_{S}||\Omega (M)-\Omega ({\mathrm{XSY}}^{T}){\left|\right|}_{F}$$

where $\left|\right|\cdot {\left|\right|}_{F}$ denotes the Frobenius norm (i.e., the sum of squared matrix entries). Note that, even though the embedded minimization is over S, the cost function still depends on the matrices X and Y. The idea is precisely to identify an optimal projection subspace, by finding an orthogonal X and Y, such that it minimizes the discrepancy between the observed entries of M and the corresponding entries of its low-dimensional, linearly-projected model $\hat{M}$. The observed entries $\Omega (\hat{M})$ are an as faithful as possible reproduction of the observed entries Ω(M). Eventually, through the optimization of F(X,Y), we also determine *all other entries* of $\hat{M}$, including the entries $℧(\hat{M})$, which can serve as repaired or filled-in values for the corrupted entries ℧(M).

The issue, however, is that there are several unknowns. To begin with, the rank *r* of the matrix $\hat{M}$, which determines the size of S, is unknown. Second, optimizing the aforementioned cost function would be a tough undertaking with no prior assumptions about the missing entries. A solution comes from defining a new matrix $M^{E}$:

$$M_{\mathrm{ij}}^{E}=\left\{\begin{array}{cc} M_{\mathrm{ij}}, & \mathrm{if}\;\{i,j\}\in \Omega (M)\\ 0, & \mathrm{otherwise} \end{array}\right.$$

Thus, the corrupted entries of ℧(M) are simply replaced by zeroes. A low-rank matrix closest to this zero-filled matrix $M^{E}$ is easy to find with singular value decomposition (SVD) and provides a good first guess for the rank *r* of $\hat{M}$ and S, which further needs to be adjusted and optimized as an algorithm hyperparameter (see later). Furthermore, to facilitate the convergence of the optimization of F(X,Y), one can use the factors of the singular value decomposition of $M^{E}={\hat{M}}_{\left(0\right)}=X_{\left(0\right)}S_{0}Y_{\left(0\right)}^{T}$ as initial conditions. Starting from this ${\hat{M}}_{\left(0\right)}$, the matrix entries can then be adjusted via a gradient descent algorithm until a local optimum of F(X,Y) is found.

Some additional technical aspects must be taken into account to guarantee the performance, such as the “trimming” of $M^{E}$ to eliminate over-represented rows and columns and the use of a suitable regularization during the optimization. For these details, we invite the reader to refer to the original publications introducing the OptSpace algorithm and its variants [14].

**Figure 2:**
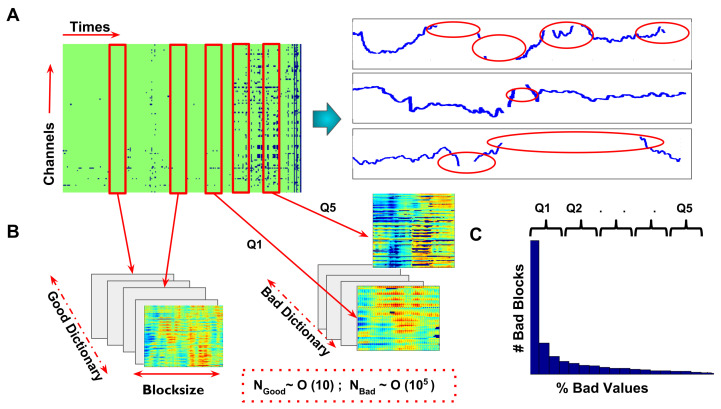
Generation of test data with artificial artifacts. (**A**) (left) Example of a typical EEG recording, including a fairly good time-segment of 20 s from one human infant. Artifact-related gaps are shown as blue entries in the data matrix. A sample short epoch with missing values due to artifacts is shown for certain example channels in the right side of panel A. The task was to numerically evaluate how well we could determine the missing entries of this matrix (highlighted in red elipses in the three zoomed-in time-series). (**B**) From the actual recording we extracted both “good” and “bad” blocks made of data epochs not including or including artifact gaps, respectively (see text for details, Red panels from the time x channel matrix in A). The good block library contained $10^{4}$ times less blocks than the bad block library, i.e., very few epochs were found without any artifacts. Bad blocks were sorted according to the severity of missing values in the block. (**C**) The distribution of severity (i.e., % fraction of corrupted values) followed a power law. i.e., for a given size, most of the blocks contained very few missing entries. Q1–Q5 represent the percentiles of this distribution and we used these percentile limits to group bad blocks into sub-classes with growing severity. Subsequently, we generated surrogate artifacted data blocks with a known ground truth signal, by overlaying actual artifact masks mediated from bad blocks on top of good blocks. We tested the artifact repair performance on these surrogate artifacted blocks, varying the block size, severity, and rank of the block matrix to be reconstructed.

**Figure 3:**
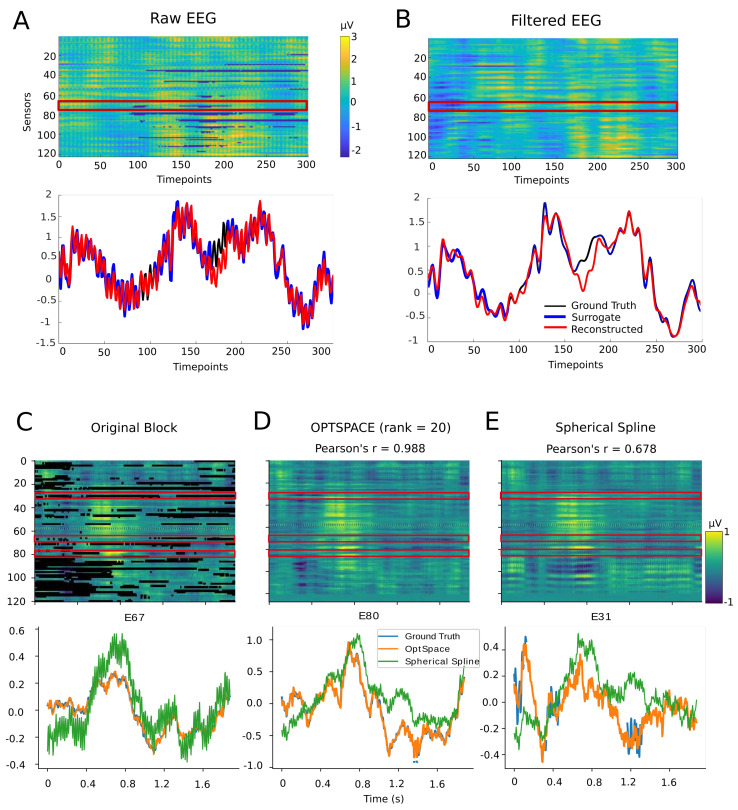
Examples of signal reconstruction. (**A**) Unfiltered simulated bad block (represented here as a C × T matrix). Missing values are visible as blue patches (bottom panel). Quality of reconstruction for a single time series obtained from one channel is represented by a red box in the top panel. Original time series (ground truth) is plotted in black, the retained sections of time series with missing entries (artificially dropped surrogate bad times) are marked in blue, with the achieved reconstructed time series in red. (**B**) (top) Reconstructed matrix and (bottom) the same channel (as in panel (**A**)) after applying a 20 Hz low-pass filter and thereby removing power-line noise. In both cases, a remarkable agreement between the ground truth (black line) and the reconstructed time series (red line) is visible. The troughs and valleys of the time series were faithfully reconstructed, even when the oscillations are strong, as before filtering, although the exact amplitude of the reconstruction might be slightly different. The performance is quantified in detail in the following: (**C**−**E**) Example comparison of OptSpace and spherical spline interpolation. (**C**). A typical block, with artifacted entries represented in black. (**D**) Block recovered by OptSpace with rank = 20. (**E**) The same block recovered with spherical spline interpolation. An apparent reversal of polarity in the lower (occipital) channels is visible. Examples of three recovered channels (highlighted by red rectangles) are shown in the bottom panels. High amplitude oscillations were over-represented in the spherical spline recovery, because it was contaminated by artifact oscillations at E59. The overall fit was better for the OptSpace algorithm (Pearson’s r = 0.988) than for spherical spline (Pearson’s r = 0.678) interpolation.

**Figure 4:**
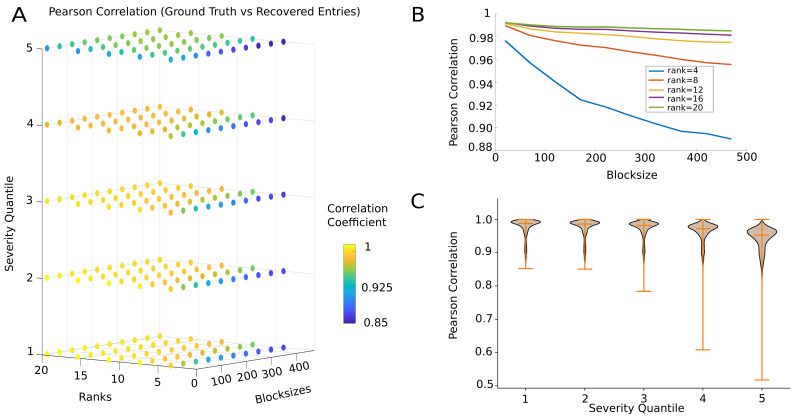
Performance benchmarking. (**A**) Effect of the three hyperparameters on the artifact repair algorithm performance. Each data point in 3-D space represents the median correlation between the actual and reconstructed signal across 500 iterations. Overall, the model fit was good, as visible from the Pearson’s correlation between the ground truth and reconstructed entries. (**B**) Combined effect of block size and rank. For very low ranks, the performance decreased exponentially with block size. However, for all r>12, the performance (Pearson’s *R* > 0.97) is good, only slightly growing with *r*. (**C**) The effect of severity across all block sizes and ranks. The violin plot gives a kernel density estimate of model performance estimated (Pearson correlation) from *N* = 25,000 iterations for each severity percentile class. When the severity of missing blocks was high, i.e., data were corrupted too much, the algorithm performed poorly on many data epochs; however, for a large fraction of blocks the overall performance remained high (Pearson’s *R* = 0.8).

Keshavan et al. [14] proved that, given the following assumptions, the described optimization procedure can recover the missing entries accurately: (1) The rank *r* of the actual matrix should be sufficiently low, with its singular values spread across all basis vectors; (2) each row and column should have at least one observed entry and there is a lower bound on the overall number of missing entries, depending on the rank and total elements of the matrix; and (3) missing values should be distributed uniformly, i.e., the algorithm cannot recover the missing entries if at some moment, all the channels were bad, or if some channels were bad at all time-points. For time series of neural activity recordings, the first two assumptions are not too far fetched, since, as previously mentioned, it is reasonable to suppose that neural activity unfolds on low-dimensional manifolds [28,29]. However, the third assumption of a uniform distribution of missing values does not always hold for EEG Data. Typical artifacts can be bursty in nature and, hence, missing values can be concentrated in a single channel or at all channels at several time points. Due to this caveat, one can only expect to *approximately* recover the true matrix. It is thus necessary to estimate how well the matrix completion works based on numerical experiments, given that the criteria for exact convergence are not always fulfilled. To accomplish this, we must create an appropriate benchmarking dataset for which the ground truth signals are known, i.e., surrogate EEG datasets with artificially introduced gaps that mimic the impact of real artifacts. In the following, we first describe the Dataset and Task Paradigm, and then describe the benchmarking method (Figure 2) as well as results obtained through benchmarking (Figure 3 and Figure 4).

### 2.2. EEG Dataset

#### 2.2.1. Subjects and Task Paradigm

**Figure 5:**
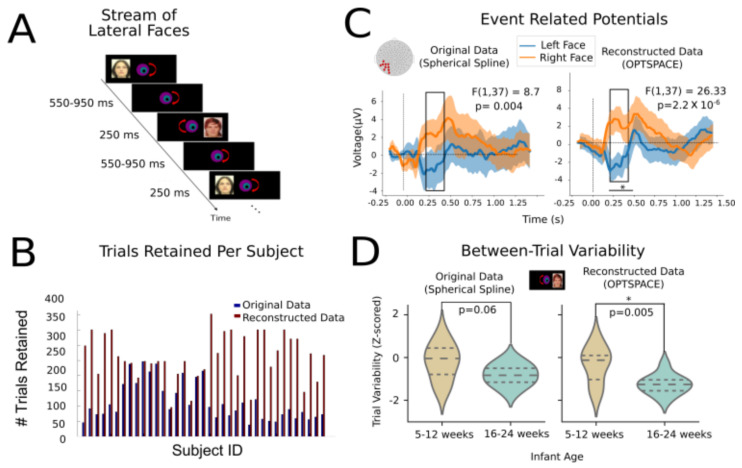
Performance of the repair algorithm in actual data analysis applications. (**A**) Experimental paradigm for evoked responses. In this simple paradigm, described in detail in [33,34], face stimuli were presented alternately in the left and right visual hemifields. (**B**) Number of sufficiently clean trials that were considered in the original data versus the actual number of trials that could be retained after artifact reparation for each of the subjects. For many subjects, the number of retained trials increased substantially, thanks to artifact reparation. (**C**) The averaged evoked response of the left occipital-temporal electrodes (position shown in the inset montage) for the two lateralized visual face stimuli (left in blue vs. right in orange), averaged across all subjects. The left panel shows the original data after spherical spline interpolation and the right panel shows data reconstructed with our matrix completion-based method.Shaded regions represent a 95% confidence interval of the mean. Typical contralateral and ipsilateral responses are visible. Our method improved the statistical power of the F-test at the peak around 370 ms. (**D**) Between-trial variability at P400 ERP components, when the faces were presented in the right hemifield for 5−12 and 16−24 week-old infants. The group difference became significant for the reconstructed data, due to a reduced across-subject variance, especially in older infants. Interquartile ranges and means of distributions are shown as dashed lines. ★ marks significant difference across condition, # = Number of trials.

The dataset serving as the basis for generating surrogates, as well as for the subsequent ERP and single-trial analyses, consisted of the electroencephalography (EEG) responses from N = 40 full-term healthy infants aged between 5.8 and 24 weeks (mean age: 14.2 weeks, 25 girls). This experiment was conducted to study the development of hemispheric specialization of face processing in human infants. The study was approved by the ethical committee for biomedical research. Parents of all infants gave written informed consent before participating in the study. Human faces were presented in the left and right hemifield, while infants fixated at the center. In order to draw infants’ attention toward the center of the screen, each trial began with a revolving colored bull’s-eye that remained there throughout the entire experiment. A variable delay between images (550 to 950 ms post-offset of the image with a 50 ms step) followed streams of face images (male or female face out of 6 neutral, unknown front-looking faces) that appeared consecutively on the left and right side of the bull’s eye for 250 ms. A minimal anticipatory glance to the left or right side was made possible by the asynchronous image presentation (variable timing between left and right faces at each trial). Figure 5A summarizes the task paradigm. Using this paradigm, we previously showed that the infants could discriminate infrequent and novel faces from the stream of repeated faces when they were present in the left but not in the right hemifield [34]. Furthermore, we also found a remarkable growth of intertrial variability and structure in this cohort [33]. Hence, here, we tried to reproduce some of these prevalidated results. The EEG paradigm is explained in detail in [34].

#### 2.2.2. Preprocessing and Artifact Labeling

For each subject, a maximum of 9–15 min of recordings (continuous or interrupted) were obtained from each of the 128 Geodesic EEG channels with a sampling rate of 250 Hz. The data were first band-pass filtered between 0.5–20 Hz, in order to remove slow drifts as well as high frequency power-line and muscle noise, resulting in a data matrix D. Additionally, the reconstruction performance was also tested without low-pass filtering. In both cases, motion and blink artifacts were marked if sudden jumps were detected exceeding a voltage amplitude >250 μV or if the deviation between fast and slow average amplitude exceeded the mark of 150 μV. Further epochs were marked as artifacted using manual inspection. When a channel had more than 70% of rejected time points, it was rejected for the entire recording (i.e., marked bad for the entire recording). On average, 5–10 electrodes were rejected for each baby (and the corresponding rows were then trimmed from the data matrix). When a time point was rejected for more than 75% of the electrodes, this time point was marked as bad for all channels (and the corresponding column was then trimmed from the data matrix). Furthermore, all entries whose voltage value was greater than 10 S.D. from the overall voltage mean were also marked as bad.

### 2.3. Numerical Analysis of Model Performance

#### 2.3.1. Hyperparameters

For any real EEG dataset, the number of total time points *T* in raw data is always much larger than the number of sensors. For our dataset in particular, the dimension of the data matrix D was $T=O\left(C^{\frac{3}{2}}\right)$. For practical reasons, it is not advisable to attempt to recover all missing entries at once in these circumstances: First, because of the large size of the input matrix (the data matrix for each subject contained ∼$10^{5}$ time points), and second, and perhaps more significantly, due to the possibility that the activity time series may visit different low-rank manifold components at different time points, due to spontaneous or evoked state changes (cf. [35,36]).

Hence, to avoid a large information loss and to meet the expectation of the original algorithm, we split the original matrix D into a series of *k* nonoverlapping epochs $M_{k}$, each of which was assumed to have some low rank *r* and a size $C\times T_{k}$, and where the length of epoch was $T_{k}∼O\left(C\right)$. The performance of the algorithm may depend on the choice of $T_{k}$ and thus had to be tested against its variations.

Moreover, the performance also depended on our guess for the rank *r* assumed for the completion of each shorter block $M_{k}$. As previously mentioned, a good guess for this may be the rank of the zero-filled block matrix $M_{k}^{E}$. If such an estimation can provide an initial indication of the range of ranks to investigate, it could also vary substantially from block to block, due to varying noise levels more than to the actual signal dimensionality changes. We therefore made the practical choice of using the same *r* for all the different $M_{k}$’s into which D was split. We, thus, treated *r* as a second hyperparameter, whose variations may affect performance and should be optimized.

Finally, the performance of the algorithm also depends on the severity of the artifacts to repair, i.e., on the number of corrupted entries within $℧\left(M_{k}\right)$. When benchmarking the algorithm, the severity percentile class *q* can also be chosen, as we can generate surrogate artifacted blocks by overlaying on a good block $M^{\left(good\right)}$ the artifact mask $B\left(M^{\left(bad\right)}\right)$ of bad blocks with the desired artifact severity (see next subsection).

#### 2.3.2. Library Construction and Bootstrapping

In order to generate realistic surrogate data for benchmarking, we first built a library of good and bad data chunks, as follows: According to the previously introduced definition, we call ℧(D) the set of corrupted entries of D, and Ω(D) its complementary set of good entries. We call any matrix block formed by a set of temporally contiguous columns of D with entries all belonging to Ω(D) a “good” block (i.e., without artifacted entries). Conversely, a matrix block formed by a set of temporally contiguous columns of D with at least some entries belonging to ℧(D) is a possible “bad” block (i.e., with artifacted entries). Parsing through all subject’s continuous EEG data files, we built a collection of all possible good and bad blocks, which we call good and bad libraries. Example good and bad blocks from an actual EEG data matrix are shown in Figure 2A,B. The *artifact severity* of a bad block is evaluated as the percent fraction of block entries that are corrupted. The distribution of the artifact severity in the library of all bad blocks can be computed, and blocks can be divided based on severity classes.For this, we calculated five severity levels based on quantiles of the % bad value distribution (cf. Figure 2C), i.e., we divided the bad blocks in five equiprobable classes, based on how severely they were corrupted by artifacts. We finally also defined a M∈bad(D), *artifact mask* for each bad block, computed as:

$$B\left(M\right)=\left\{\begin{array}{cc} 1, & \;\mathrm{if}\;i,j\in \Omega (M)\\ 0, & \mathrm{otherwise} \end{array}\right.$$

In every iteration, a chunk of EEG data of a given block size was selected at random from any of the subjects in the dataset, without replacement. The size of the chunk was systematically varied between a maximum number of $T_{k,max}=470$ time points (or 1.88 s) and a minimum $T_{k,min}=20$ of time points (or 0.08 s). This randomly chosen block was labeled good or bad depending on whether it included artifacted entries or not. This random extraction of good and bad blocks continued until we reached the desired library size. This procedure is illustrated in Figure 2B. The number of good blocks found depended on how noisy the EEG data were. For instance, in our dataset, for the data table D for one subject, we could extract a ratio of ∼20 good blocks vs. $\;O(10^{5})$ bad blocks when using the largest block length $T_{k,max}$. A larger number of good blocks were found when constructing libraries with shorter block sizes (as it was easier to fit shorter blocks between two artifact-induced data gaps). For every block size, we also assigned each of the extracted bad blocks to a percentile class of severity *q*. Note that, even for $T_{k}=T_{k,max}$, most of the bad blocks had only a few corrupted entries, as indicated by the strongly right-skewed distribution of artifact severity in the extracted library bad(D), as shown in Figure 2B.

Based on the libraries good(D) and bad(D) of good and bad blocks extracted from the considered data table D, we generated realistic and data-compliant surrogate bad matrices. For each block size value $T_{k}\in$ {20, 70, 120, …, 470} and severity percentile q∈ {1, 2, …, 5}, good blocks $M^{\left(good\right)}$ and bad blocks $M^{\left(bad\right)}$ were sampled with replacements from good(D) and bad(D), respectively. A surrogate artifacted block $M^{(good),E}$ was then computed by performing entry-wise multiplication between the original good block $M^{\left(good\right)}$ and the artifact mask $B\left(M^{\left(bad\right)}\right)$ of the bad block $M^{\left(bad\right)}$. In this way, we obtained a surrogate artifacted block for which the ground truth signal was masked by the imposed artifact pattern but that could be recovered for comparison, i.e., $℧(M^{\left(good\right)})$ is empty by definition.

Each of these corrupted matrices were used as input to the matrix completion algorithm and the performance was measured in terms of both the Frobenius distance and Pearson correlation between the ground truth good matrix block $M^{\left(good\right)}$ and the matrix with repaired gaps generated from $M^{(good),E}$. Completion was performed for any block-size $T_{k}$ and severity percentile, by varying systematically the chosen fixed rank values r∈ {4, 8, 12, …, 20}. Overall, the performance of completions was studied through performing n=500 bootstrapped iterations for each combination of block size, severity, and rank. The results for these experiments are presented in Figure 3 and Figure 4 and discussed in Section 3.

### 2.4. Method Validation in Real Data Analysis Applications

No method can create information, and our method is no exception. This algorithm does, not only fill the missing values with values that may be different from the real ones, but furthermore can alter the time series, even in the sections where real data are available, because the original data matrix with gaps is replaced by a low rank approximation without gaps. It was thus important to understand whether our artifact repair algorithm can guarantee or, ideally, improve the quality of real neuroscience-relevant analyses with respect to analyses without completion or those completed with competitor state-of-the-art techniques. Hence, we quantified the event-related potentials (ERPs) and modulations of between-trial variability quenching (VQ)—two relevant markers for cognitive neuroscience analyses— and assessed the relative improvement achieved by the two data completion methods (our proposed method and an alternative, more conventional, spherical spline interpolation method). Spherical spline interpolation is a spatial interpolation method that is applied for filling missing entries in the bad channels of a subject’s evoked responses (e.g., using MNE Python’s built-in function evoked.interpolate_bads) [37]. At each time-point, spline interpolation considers the relative spatial positions of the good and bad channels on the head model. The voltage entries of all the good and bad channels are first projected onto a unit sphere. Then a matrix is computed, to project good channels onto the bad channels on this sphere, which is then used to interpolate data in the bad channels. A detailed explanation of this method can be found in [32]. Importantly, this method does not explicitly take into account temporal correlations in the data and was not designed to repair small gaps, but operates by generating a full spline-interpolated time series for an entire channel marked as bad. A performance comparison between our method and spherical spline interpolation is given in Figure 3 and Figure 5. For a more robust comparison, we chose 300 randomly selected surrogate bad blocks and applied either optspace or spherical spline interpolation (after marking any channel containing artifacted values as “bad”). We then compared the correlation between the original, ground-truth block and the reconstructed blocks with the two methods. To test if our method could reconstruct the blocks significantly better than spherical spline interpolation, we applied a right-tailed Wilcoxon rank sum test to the two Pearson correlation values for each block.

### 2.5. Event Related Potentials

We applied our reconstruction algorithm to N=34 subjects, a subset of the previously described dataset with the block size = 120 and varied ranks depending on the amount of corrupted entries in the blocks. After signal reconstruction with both methods, baseline correction and reference averaging steps were performed to improve the signal-to-noise ratio. Channels were further rejected if they remained irrecoverable after applying OptSpace throughout all epochs and were normalized using the global field power (GFP) at each time point [38]. We then averaged the evoked responses from the left occipital-temporal cluster across time-aligned trials and compared the resulting ERPs when visual face stimuli were presented in either the left or right visual hemifield. A good ERP response was computed by further averaging across subjects. Subjects for which the data quality was marked as insufficient in the original cohort (without reconstruction) were removed from further comparisons (Figure 5A represents the experimental paradigm, described in greater detail by [34]). The comparison between ERPs obtained after data reparation with the two considered methods is discussed later and depicted in Figure 5C.

### 2.6. Between-Trial Variability

Recently, our group and others have shown that the single-trial variability of a subject can serve as an important marker of cognitive development and flexibility in infants and children [33,39]. Indeed, even if part of the between-trial variability could be due to unwanted environmental and biological noise, when these noise sources are sufficiently removed, the remaining between-trial variability can reflect underlying dynamical changes [40]. Such between-trial variability is quenched at the onset of stimulus, showing a nonlinear relationship between stimulus-evoked and resting-state neural activity. A growing number of studies have proven that this measure is a relevant marker of task-driven or subject-driven trial-by-trial fluctuations in performance and attention ([41,42,43]). Hence, here, we considered how our artifact reparation procedures can improve the quantification of between-trial signal variability, beyond ERP analyses. Specifically, we focused on neural trajectory variability in a time range matching the P400 response (i.e., a 400–600 ms peristimulus time window), which displays a stronger maturation of “event related variability” (ERV) with age during early infancy (see [33] for details). To evaluate the between-trial variability, we calculated, (following [33]), pairwise correlation distances between different trials at each time point and averaged these distances over the time range of interest for each subject. We then compared this metric between very young (5–12 weeks old) and older (16–24 weeks old) infants, first with the original data (with spline interpolation) and, second, using low-rank matrix completion signal reconstruction (cf. Figure 5D).

## 3. Results

### 3.1. Algorithm Performance Benchmarking with Surrogate Artifacted Data

As described in the previous Methods section, we generated a library of ∼$10^{5}$ realistic patterns of artifact-induced data corruption and generated a a similar number of artificially artifacted data blocks, to test how well our matrix completion algorithm could reproduce the ground truth. Typical examples of reconstructed time series are shown in Figure 3, obtained using an artifact mask belonging to the fourth percentile of severity. Figure 3A shows the simulated bad block structure for a block sampled from the fourth percentile (matching the example bad block shown in Figure 2A). In Figure 3A,B we show the actual and reconstructed time series for a representative artifacted channel. The reconstructed time series, shown in red color, recovered the prominent oscillations displayed by the ground truth data in a pretty faithful manner (in black and blue). Notably, oscillations were regenerated even within the artificially punched-in artifact-induced gaps.

The libraries of good and bad blocks were generated directly from the original data, without applying any filtering, and hence power-line noise was present in the channels, which is visible in panel A. The fitting improved remarkably when the power-line noise was filtered out using a 20 Hz low-pass filter. Figure 3B shows the reconstructed matrix and signals after filtering, and we can observe that the temporal trends still appeared to be well-recovered, even after filtering. Note that we obtained better reconstructions by applying matrix completion first on the signal unfiltered for line noise and then afterwards filtering the reconstructed signal, rather than performing matrix completion on a prefiltered signal. This probably occurred because line noise contributes to the increase in the spatiotemporal redundancy among channels, thus reducing the signal rank. In Figure 3C–E we compare the rendering quality achieved by our OptSpace-based algorithm with the state-of-the-art spherical spline method. To do so, we randomly selected 300 simulated bad blocks with maximum block sizes (i.e., ∼470 time-points) from the 4th severity percentile. Then we applied spherical spline interpolation, as described in the Method’s section. The representative example shown in Figure 3C–E clearly demonstrates that the OptSpace algorithm performed better in recovering the ground truth entries than the spherical spline interpolation. In the shown example, the spherical spline-interpolated time series tended to be systematically shifted with respect to the original time series. This is because the spherical-spline method seeks for missing information only through space, i.e., regenerating the bad channel through operations on the signals of spatially neighboring channels. On the contrary, OptSpace regenerates local missing sections of the artifacted channels seeking for information across both space *and* time, and maintaining signal continuity at each of the repaired gaps (see Section 4). Overall, considering the comparisons over the full test ensemble of bad blocks, Pearson’s correlation between the ground truth and reconstructed missing entries was significantly higher for OptSpace than for spherical spline interpolation (Wilcoxon signed rank test $z\left(299,1\right)=45145,p=3.1\times 10^{-51}$), suggesting that the bad blocks were reproduced significantly better by the OptSpace algorithm than the competing spherical spline algorithm.

In Figure 4, we evulate the actual quality of reconstruction—quantified in terms of the Pearson correlation distance between the actual and regenerated signals; achieved by the algorithm as a function of the different hyperparameters affecting performance: block size, projection rank, and severity percentile of the applied artifact masks. The accuracy reported here was O($10^{4}$) across all iterations, including all severity quantiles, ranks, and block sizes.

Overall, the model performed well across all bootstrapped simulations (median correlation between all matrix entries = 0.976±0.0338, median Frobenius distance = 0.107±0.0685). The observed entries were recovered nearly perfectly (median correlation = 0.997±0.01, median Frobenius distance = 0.037±0.036). Apart from a few data blocks (10% blocks out of O(104)) where it was impossible to meet the assumptions of the algorithm, all other correlations between recovered entries and ground-truth entries were positive. The accuracy of the recovered artifacted entries was less than that of the revealed entries, but it was still significantly high (median correlation = 0.47±0.36, median Frobenius distance = 0.35±0.25). In all cases, the correlations (and Frobenius distance) were significantly higher (smaller) than is expected using chance level reconstruction (estimated via permutation testing, deeming correlations significant with 95% confidence when larger than $C_{chance}=0.03$ and distances when smaller than $D_{chance}=39.038$).

### 3.2. Dependence of Algorithm Performance on Algorithm Hyperparameters

The variability of the accuracy scores may have depended on the choice of hyperparameters. We then moved to study the dependence of the repair performance upon the tuning of the three hyperparameters.

Figure 4A presents the performance in a 3-D parameter space spanning all hyperparameter combinations. The hyperparameter space can be clearly divided into different regions, according to the median model performance. Specifically, close to an exact recovery (r>0.99) was possible up to the third percentile of artifact severity, when using smaller a block size and higher rank values. The average reconstruction accuracy remained satisfactorily high (*r*∼0.88) even for the highest severity percentile, i.e., when 30–45% of the entries were corrupted.

The performance decreased with increasing block size. However, this decrease was much less marked when a sufficiently large rank was used; i.e., as Figure 4B suggests, there was a combined effect of rank and block size. For the lowest rank r=4, the model performance decreased quite rapidly with block size. However, for the rank r≥12 onward, there was no significant gain in performance from further raising the rank. This may be an indirect confirmation that our initial low-rank assumption for the data was well justified (see Section 4). Indeed, the median rank of the data blocks before applying the OptSpace algorithm were quite high (median rank =114±10), and reflected the number of channels with nonzero entries, suggesting that the higher matrix rank reflects the noise in the data. This was further confirmed by looking at the same relationship, specifically for the recovery of missing values. As observed in Supplementary Figure S1, the accuracy in missing value recovery increased (or the error in reconstruction decreased) with the increase in block size, for all approximated matrix ranks. This was due to the fact that the longer block sizes tolerated more deviations from the actual neural trajectory, and hence the local reconstruction improved at the expense of the overall global reconstruction of the manifold. Here again, lower matrix ranks could approximate the missing entries better than higher ranks, again suggesting that the lower matrix rank reduced overfitting of the existing entries, allowing for better reconstruction of the missing entries.

Finally, Figure 4C displays the effect of artifact severity percentile on performance. For all severity levels, our method appeared to be capable of reasonably reconstructing the ground truth, with a greater than 90% mean accuracy. However, as revealed by a developing a downward performance distribution tail, as the severity quartile increased, some of the artifacted blocks began to be reconstructed with reduced accuracy. With increased severity, the reconstruction of missing values was indeed compromised (cf. Supplementary Figure S2), i.e., artifacts in the first quantile were more faithfully reconstructed (median Pearson’s r = 0.82) than those in the fifth quantile (Pearson’s r = 0.4). However, for all severity classes, the performance remained significantly higher, as indicated by the positive correlation coefficient values. This trend was not as marked for the revealed entries, as they were always reconstructed almost perfectly. The effect of severity on recovering artifacted entries can be understood in terms of the violation of a random sampling of the missing values. As the severity of artifacts increased, there was insufficient information in the revealed entries of the matrix to faithfully reconstruct the missing entries. Furthermore, the chance of witnessing bursts of missing values concentrated in a single channel or clustered around single epoch of time grows rapidly with the increase in severity, further lowering the quality of reconstruction.

### 3.3. Event-Related Potentials

Using the optimal values for the block size and rank hyperparameters, as determined through the benchmarking process, we applied signal reconstruction to an actual EEG dataset derived from a real cognitive neuroscience experiment [34] and tested how the reparation of real (rather than imposed) artifact gaps affected the extraction of event related potentials (ERPs) and their comparison between trial groups (Figure 5).

Figure 5A summarizes the experimental paradigm. Visual face stimuli were presented alternately in the left and right visual hemifields. This task was performed as previously described in the Methods by very young infants, an atypical subject cohort, for which the level of corruption by artifacts in a time series is particularly large, forcing the discarding of a large number of experimental trials.

The first positive effect of using signal reconstruction is observed in Figure 5B. After matrix completion, the number of trials that reached quality levels sufficient for their inclusion in the analyses increased by a twofold factor (trials were rejected if more than 50% of the entries were corrupted).

In Figure 5B, we show a comparison between the extracted ERPs. The left panel of Figure 5C shows the ERP response from the left occipital-temporal clusters for the original data, where the gaps were reconstructed with the conventional spherical spline interpolation. Note that, even though the electrode voltage may have momentarily drifted, the spherical spline interpolation was used to replace electrode activity for the entire epoch. Hence, the spherical spline did not take into account the temporal properties, only spatial properties. On the other hand, OptSpace took into account, not only the trajectories of evoked responses, but also the spontaneous activity. Analogous ERP time courses are shown in the right panel, but these were derived from signals repaired with our low-rank matrix completion method. Two interesting observations can be made: First, the grand-average curves in both cases had excellent similarity, with the reconstructed data replicating the peaks and troughs at the exact same latency as the minimally interpolated data (with spherical spline interpolation). This is noteworthy, as our method not only fills the gaps in the evoked data but fully regenerates the entire time series to make it compliant with a low-rank model, and hence, the alterations in ERPs could even have been induced far from the epochs where most artifacts were detected. The fact that ERP was still faithfully captured indicates that the ERP spatiotemporal characteristics were well determined by the topology of the low-rank manifold over which the neural trajectory unfolded and that our algorithm learned to extract (see Section 4).

Second, only for the low-rank matrix-completed time series did we observe a significant reduction of the standard error of the mean (SEM) across subjects, due to the fact that we could include many more trials, many of which in reality where corrupted only at specific locations but carried otherwise meaningful and relevant information. As a result, the increased statistical power made possible detecting significant effects of a smaller size. Notably, we calculated the contra- and ipsilateral ERP responses of the left occipital-temporal channels when faces were presented in the left and right hemifield. The two responses significantly differed in the time window of 250–450 ms. We found that this response was significantly different depending on the hemifield in which the faces were presented. Owing to the reduced across-subject variance and increased confidence in the mean curve, we observed a 1000-fold increase in the size of this effect for reconstructed data as compared to the original data (one sample f-test F(1, 37) = 8.7, *p* = 0.004 for spherical spline interpolation, one sample f-test F(1, 37) = 26.33, *p* = $2.2\times 10^{-6}$ for low-rank matrix completion).

### 3.4. Between-Trial Variability Quenching

As demonstrated in the previous section, our method was better able to successfully reconstruct ERPs than the spherical spline method. In a previous study by our group [33], we showed that the variability of neural trajectories in response to stimuli was a sensible neuromarker for tracking cognitive development. In particular, through a paradigm that we named “event-related-variability” (ERV) analysis in [33], we showed that the between-trial variability is quenched at certain peristimulus times and that the exact timing and spatial pattern of quenching is modulated by development.

Here, we show that artifact reparation via our novel method can also improve the sensitivity of response-variability analyses. Figure 5D depicts the subject-level distributions of Z-scored between-trial variability in the vicinity of the P400 ERP component for two age groups: young infants (5–12 weeks old) and older infants (16–24 weeks). Here again, we can observe that the single-subject distributions of between-trial variability better discriminated between age groups for the reconstructed data as compared to the original data (Wilcoxon rank sum test for equality of medians between the two age groups: *p* = 0.06 for original data vs. *p* = 0.005 for reconstructed data).

Hence, we are able to show that the improved number of trials due to reconstruction did not simply improve the average discrimination due to the higher-number of included trials, but also faithfully reconstructed individual trials, so as to track differences in their variability.

Overall, our results suggest that such a method is not only able to retain a higher number of trials, but also able to retain subtle features of the intrinsic neural signals.

## 4. Discussion

In this study, we have demonstrated how a low-rank matrix completion algorithm along with gradient descent learning of Grassman manifolds can be used to “fill’’ the missing entries due to artifacts in real EEG recordings. After comparing this algorithm’s performance with a state-of-the-art alternative method and studying the performance dependence on selected parameters, we also provided a proof-of-concept demonstration of the algorithm’s usefulness, showing how the ERP and single-trial analyses of an actual EEG dataset can be improved. In particular, we have shown that the use of our method can substantially increase the number of trials that can be retained for subsequent analyses, as compared to a simple spherical-spline interpolation, thus generally increasing the statistical power of estimation and discrimination. Our method can easily be inserted within existing preprocessing pipelines in the case of cohorts for which the noise is high and yet where trial retention is an absolute necessity.

Other methods for artifact repair have been introduced. With a few notable exceptions (cf. [44]), most of the signal recovery algorithms for neural data rely either on channel-by-channel recovery of signals using algorithms developed for time-series interpolation [1] or use spatial interpolation at specific time points [45]. These approaches do not take advantage of the redundancy of the EEG signal in the spatiotemporal and spectral domains. The spherical spline spatial interpolation method used here as comparison belongs to this category. Other methods previously described in the literature, however, use similar approaches to ours and were thus particularly adapted for the reparation of sparse artifact damages. For instance, the STAR method by [46] also adopts a linear projection strategy, by attempting to estimate missing sections of the data in terms of the linear covariance structure of data from observed channels. There are two key differences with our method. First, in our approach, we perform local projections on short chunks of the data, rather than on the whole time series. In other words, the linear model for projection is locally adjusted, rather than globally determined, so different projection matrices can eventually be used for different individual gaps within the same recording. Such local adjustment could, however, be implemented in the STAR method; estimating different covariance matrices for different data segments. A second, more fundamental difference is our emphasis on low-dimensionality. In a covariance-based approach such as STAR, the underlying completion model always has the same dimensionality of the original data. In our case, on the contrary, we explicitly assume that the right completion model has a smaller dimensionality than the recorded dataset. This is a strong assumption, potentially prone to information loss. The justification of our low-dimensionality ansatz is not of a methodological nature, but reflects a prior hypothesis motivated by experimental findings.

Indeed, a growing amount of literature shows that neural signals become redundant due to high spatiotemporal correlation of the spontaneous activity observed at many different scales [28,29]. This reflects the fact that neural trajectories unfold over low-dimensional brain-state-specific manifolds, due to both learning and the self-organized collective nature of brain network dynamics [22,24,26]. The local geometric structure of neural activity manifolds thus constrains the way in which neural activity configurations can fluctuate, and effectively reduces the dimensionality of the space in which these configurations can be sampled. Our method takes advantage of this hypothesis by learning the local tangent space orientation of the unknown underlying manifold sampled using the multi-variate EEG time series. Hence, unlike other interpolation methods, our method does not simply fill the missing entries when artifacts are detected, but completely regenerates a "fake" data matrix under the local low-dimensionality ansatz. We have checked, however, that, for the observed entries, the correlation between the actually measured activity values and the regenerated ones was nearly perfect (cf. Supplementary Figure S2B). This means that the low-dimensionality ansatz is not completely “out of the blue sky” but correctly embeds an essential aspect of the actual data. By constraining the regeneration of missing entries, to be tested on locally adjusted linear spaces tangent to the neural trajectory manifold, we automatically perform a denoising operation, as noise causes trajectories to transiently depart from the manifold. In this sense, the reduced but still significant performance of rendering for “hidden” (i.e., surrogate missing) data matrix entries could be explained by the fact that the local high-dimensional noise component of the signal cannot be regenerated, only for its low-dimensional trend.

With regard to the actual EEG data analyses discussed by Figure 5, it may seem surprising that the result quality can actually be improved. Indeed, because of data processing inequality, no algorithm, independently from how powerful and sophisticated it is, can recreate permanently lost information. A solution to this apparent paradox may be that the irremediably lost information conveyed by artifacted data sections is *redundant* with information present in some other observations, either the activity in other channels or even the same channel at different times outside the corrupted sections. Due to redundancy, this information is equivalent to the lost information. However, because of the technical difficult of handling artifacted channels, too much data are usually discarded, thus throwing away usable “copies” of the missing information. Ultimately, signal repair allows keeping more trials. Besides the fact that this increased sample size boosts statistical power per se, it does this in a reliable and sound manner, as the new information injected in the analysis by the additional included trials is genuine and not only a random guess. This is also the reason why, not only the ensemble-level ERP analyses of Figure 5C but also the “ERV” analyses [33] of Figure 5D can benefit from signal repair. Our procedure is not limited to inferring a generic trial-group level signal trend common to multiple trials (which would be sufficient to improve ERP estimation, but not single-trial-level analyses). On the contrary it actually infers information about activity within gaps at specific spatiotemporal locations, extracting it from redundant copies at different spatiotemporal locations. In other words, each trial can be individually regenerated to a certain extent, and not only the average activity. This is a crucial asset of our method, opening the way to its application in studies in which the functional role of between-trial variability is explicitly investigated [33,42,47,48].

Here, we illustrated only one proof-of-concept application of our method to a high-density EEG dataset, including the first semester of the life of infants [33,34]. However, given the generality of this method, it could be applied to any multisensory neural data, including LFP, MEG, and ECoG, to name a few. However, the optimal hyperparameters for selection could be different for different datasets and types of signal. In the case of our example application, we estimated optimal parameters based on a bootstrapping library of surrogate artifacted data, which was highly tailored to the specific dataset we wanted to complete. Precisely the same procedure could be adapted to other datasets, for different scientific problems and different modalities of recording, thus opening the way to a dataset-specific optimization of repair procedures; potentially superior to benchmarking with simulated signals from generic statistical models (cf. e.g., [46]).

### Limitations and Future Work

One of the main limitations of our OptSpace-based method is its time complexity. Unlike most interpolation techniques, this method is applied not only to the peristimulus epoch times, but on the entire data matrix of continuous data, artificially segmented into blocks of reasonable sizes. Such a “holistic” approach is needed, as we need all data to infer a bundle of hyperplanes locally tangent to the hypothesized but unknown neural activity manifold. In our procedure, an optimization step is iterated as many times as the number of time blocks in which the continuous multivariate time series is divided. While multiple iterations are needed to improve the accuracy of the inference of missing values, each of these optimization steps scale linearly with the number of missing entries |E|, rank *r*, and blocksize *n* as time complexity is O(|E|rlogn). This means that a typical run of the OptSpace algorithm with 10–20 min of EEG recording took approximately 10–15 min of processing per subject, with a moderate-to-high number of missing entries on MATLAB. Since the computational cost of applying our data repair method is not negligible, one should consider beforehand what is the expected level of improvement that its application could yield for the planned data analyses. When studying, for instance, data from healthy adults in highly stable experimental conditions, the gain from applying data completion could be so low that the additional time (and CO_2_) expenditure needed for signal repair may not be worth undertaking. There are cases, however, such as studies involving patients with Parkinson’s or Alzheimer’s diseases and elderly subjects, or young children and infants, in which increasing trial retention is an absolute necessity, as the degree of signal corruption due to artifacts is very high. It is, thus, especially for these “atypical cohorts” that we expect our method will serve as a valuable resource.

We note that low-rank matrix completion is a machine learning optimization problem, whose range of applications goes well beyond signal repair. This means that the very active research in other research fields— e.g., compressed sensing [28]—may yield superior and faster algorithms for matrix completion in the near future. As our pipeline was not built ad hoc for EEG signal repair, but capitalizes on the encounter between a general hypothesis—low dimensionality and activity—and an equally general and widespread algorithmic problem, it will be possible to profit from any advances obtained in different fields, simply by replacing the OptSpace step with another better algorithm performing the same low-rank matrix completion task. For example, the time limitation might be mitigated by making use of faster implementations of matrix completion methods, such as fancyImpute (available in R and Python ([49])), which are optimized for parallel processing. Our benchmarking results suggest that such matrix completion algorithms are compatible with use with EEG data.

## 5. Conclusions

A low-rank matrix completion method was able to successfully repair artifacts, without any assumptions about the underlying sources that might have generated these artifacts. Taking advantage of the combined spatiotemporal structure of the neural data, this method was able to successfully reconstruct the signal of interest, not just for the evoked activity, but also for intrinsic neural activity. This method significantly improved the trial retention, which in many cases further improves the effect sizes during hypothesis testing. Such a method improves the usability of noisy EEG signals, a critical aspect, especially when the number of rejected trials tends to be large, as in atypical and pathological cohorts.

## Data Availability

Data will be available upon reasonable request to Dr. Ghislaine Dehaene-Lambertz.

## Supplementary Materials

The following supporting information can be downloaded at: https://www.mdpi.com/article/10.3390/s23104847/s1.
