# Supplementary material for: Repairing Artifacts in Neural Activity Recordings Using Low-Rank Matrix Estimation
Source: Sensors (Basel). 2023 May 17;23(10):4847. doi: 10.3390/s23104847 (PMC10220667; doi:10.3390/s23104847)
Supplement: Supplementary file 1 [file sensors-23-04847-s001.zip › sensors-2366599-supplementary.pdf]

Supplementary Figures

Supplementary Figure S1

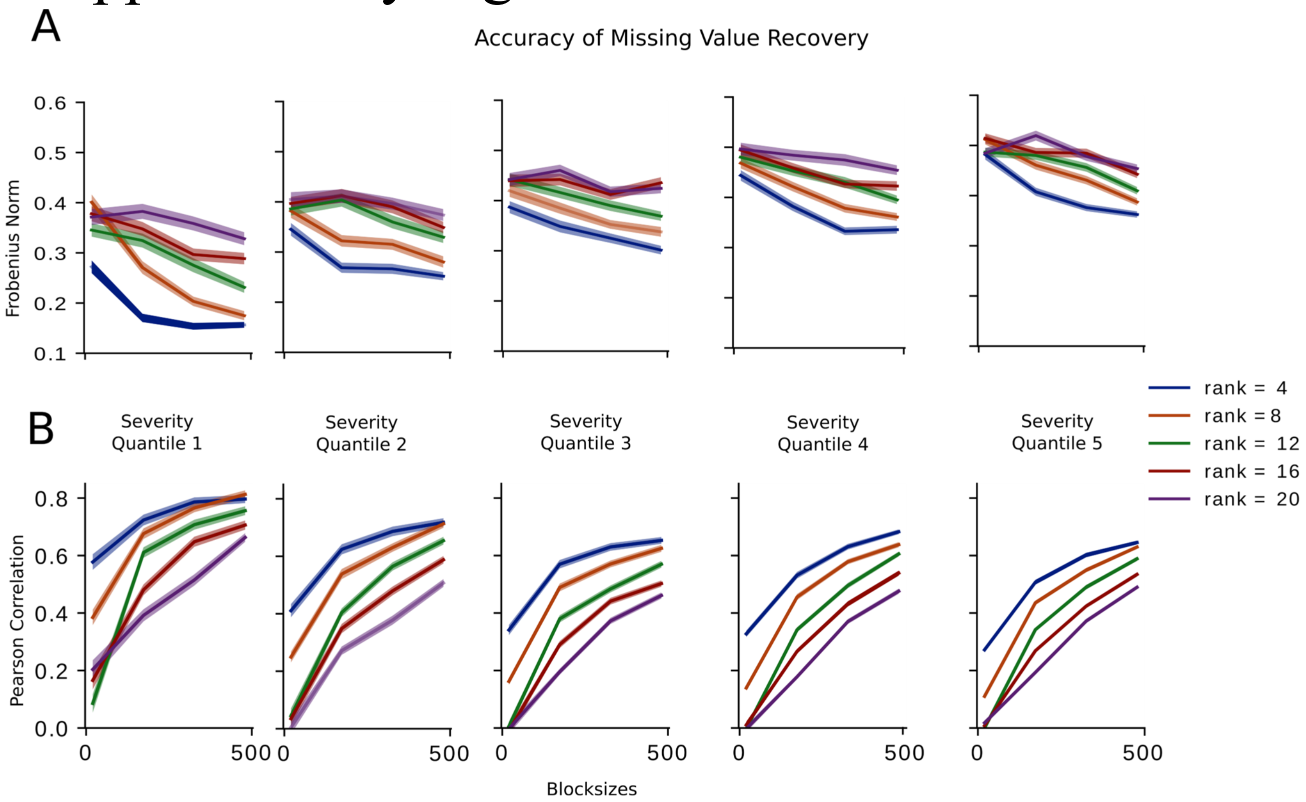

Figure S1: Summary of model accuracy for recovering missing entries. **(A)** Frobenius norm and **(B)** Pearson correlation coefficients between ground truth and simulated blocks, sorted by severity quantiles. Shaded regions represent the standard error of the mean across 500 simulated bad blocks for each condition (each curve). Overall, the increase of rank includes noisier entries, reducing the recovery accuracy for missing entries; however, this performance increased when increasing~block sizes.

Supplementary Figure S2

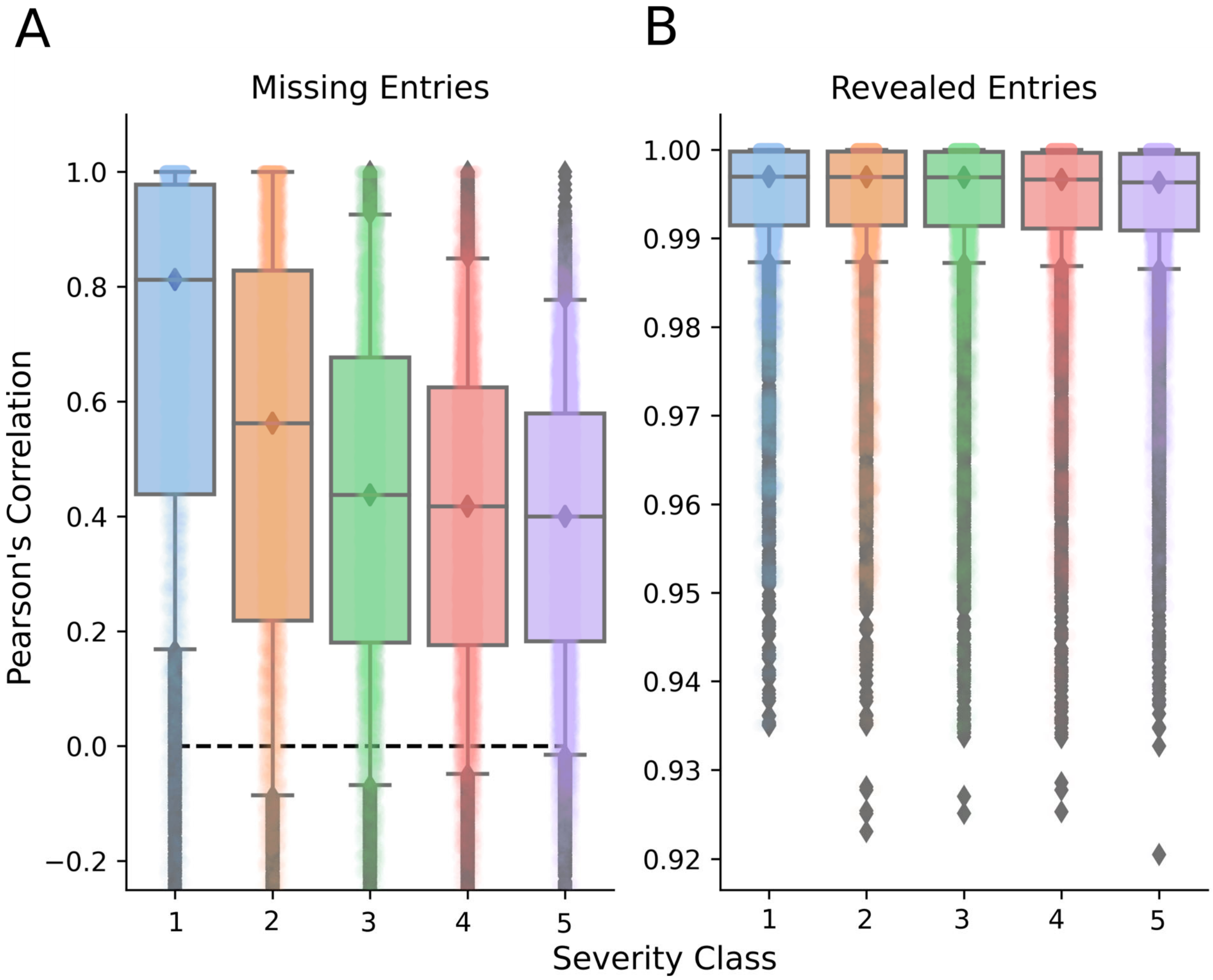

Figure S1: Effects of severity on model performance. Boxplots represent the distribution of Pearson's correlation coefficient between ground truth and the recovered entries by OptSpace across all 25,000 simulations, for when **(A)** the entries are missing from bad blocks (i.e., artifacted) versus **(B)** when the entries were revealed. The performance in recovering missing entries reduced with increased severity, while for the revealed entries, this remained constant. Nonetheless, the correlation remained significantly higher than zero across all entries. The transparent dots represent actual data points.
